# Socio-economic and regional variation in breast and cervical cancer screening among Indian women of reproductive age: a study from National Family Health Survey, 2019-21

**DOI:** 10.1186/s12885-022-10387-9

**Published:** 2022-12-07

**Authors:** Soumendu Sen, Pijush Kanti Khan, Tabassum Wadasadawala, Sanjay K Mohanty

**Affiliations:** 1 Department of Population and Development, International Institute for Population Sciences, Mumbai, India; 2 International Institute of Health Management Research, Delhi, India; 3 Department of Radiation Oncology, Advanced Centre for Treatment, Research and Education in Cancer (ACTREC), Tata Memorial Centre, Homi Bhabha National Institute, Mumbai, India; 4 Department of Population and Development, International Institute for Population Sciences, Mumbai, India

**Keywords:** Breast cancer, Cervical cancer, Screening, Women, NFHS, India

## Abstract

**Background:**

In India, breast and cervical cancers account for two-fifths of all cancers and are predominantly prevalent among women in the reproductive age group. The Government of India recommended screening of breast and cervical cancer among women aged 30 years and over. This study examines the socio-economic and regional variations of breast and cervical screening among Indian women in the reproductive age.

**Methods:**

A full sample of 707,119 women aged 15–49 and a sub-sample of 357,353 women aged 30–49 from National Family Health Survey-5 (2019-21) were used in the analysis. Self-reported ever screening for breast and cervical cancer for women aged 15–49 and women aged 30–49 were outcome variables. A set of socio-economic and risk factors associated with breast and cervical cancer screening were used as the predictors. Logistic regression was used to understand the significant correlates of cancer screening and, concentration index and concentration curve were used to assess the socio-economic inequality in breast and cervical cancer screening.

**Results:**

The proportion of breast and cervical cancer screening among women aged 30–49 were 877 and 1965 per 100,000 women respectively. Cancer screening was lower among women who were poor, young, had lower educational attainment and resided in rural areas. The concentration index was 0.2 for ever screening of breast cancer and 0.15 for cervical cancer among women aged 30–49 years. The concertation curve for screening of both breast and cervical cancers was pro-rich. Women with higher educational attainment [OR:1.46, 95% CI: 1.31–1.62], aged 40–49 years [OR:1.35; 95% CI: 1.28–1.43], resided in the western [OR:1.62; 95% CI:1.4–1.87] or southern [OR:6.66; 95% CI:5.93–7.49] region had significantly higher odds of up taking either of the screening. The pattern of breast and cervical cancer screening among women aged 15–49 was similar to that of women 30–49.

**Conclusion:**

The overall proportion of cancer screening among women in 30–49 age group is low in India. Early screening and treatment can reduce the burden of these cancers. Creating awareness and providing knowledge on cancer could be a key strategy for reducing the burden of breast and cervical cancers among women in the reproductive age in India.

**Supplementary Information:**

The online version contains supplementary material available at 10.1186/s12885-022-10387-9.

## Introduction

Globally, an estimated 19 million people were living with cancer in 2020 [1]. The Global Burden of Disease study estimated that cancer caused 213.2 million disability-adjusted life years (DALY) in 2016 of which 98% were years of life lost (YLL) [2]. An estimated 712,758 women and 679,421 men in India were diagnosed with cancer in 2020. The incidence rate of cancer was 104 per 100,000 women compared to 94 per 100,000 among men [3]. Breast and cervix are the two most common cancer sites for women. These two cancers account for two-fifths of all cancer cases among Indian women [3, 4]. The incidence of breast cancer in India is lower than in most of the developed nations possibly due to the lower screening rate [5]. Those who are diagnosed with cancer are diagnosed in the advanced stages, leading to a higher premature mortality [6] and pushing households into the medical poverty trap [7]. According to a report by the National Cancer Registry Programme, the age-adjusted incidence of breast cancer in India is higher in the metro cities and urban areas, whereas that of cervical cancer is higher in the north-eastern regions [8].

Studies conducted in developed countries have suggested early detection of malignancy and early start of the treatment as an essential strategy to improve disease prognosis and lower the mortality risk and excess healthcare burden [9, 10]. Studies in low and middle income countries (LMICs) including India, have found that lack of awareness, social stigma, familial negligence, inefficiency in the referral pathways, lack of essential health infrastructure in regional centres, incomplete treatment and inadequate follow-up are the major contributing factors to the low screening rate, late detection, and high mortality due to cancer [11–13]. Despite growing cases of breast and cervical cancer, effective and accessible screening programs is very limited in LMICs. Age is an important risk factor for breast and cervical cancer. With limited resources, many countries have adopted varying age for screening of breast and cervical cancer. For instance, the minimum recommended age for breast cancer screening in Vietnam is 20 years while it is 30 years in India, 35 years in Sri Lanka and 40 years each in China and Pakistan [14–17]. In the case of cervical cancer, China recommends 18 years as the minimum age of screening, while it is 20 years in Korea, 30 years each in India and Indonesia, 35 years in Thailand [18]. Despite these guidelines, the screening prevalence is low. For instance, the screening of cervical cancer varies from 7.3% in Indonesia to 22.3% in India. Among others, lack of knowledge, demographic and socio-economic-cultural, structural barriers are the factors for low screening in LMICs [19].

Of the 1.4 billion population of India in 2021, 20 million are women aged 30 to 49 years accounting for 14% of India’s population. Similarly, women aged 15 to 29 years accounts for 12% and 10% are 50 years and above. [20, 21]. Women are vulnerable section of the population, being disadvantaged both economically and socially, and bear a higher burden of disease [22]. In the reproductive age, they experience pregnancy, child birth and its complications, menopause and other morbidities [23]. Now, women in India are increasingly engaged in productive work [21].

The burden of cancer among women is growing in India and is likely to increase in the future [24]. Breast and cervical cancers are unique, in that they are mostly women specific and disproportionately affect women in the reproductive and economically productive age group. These cancers account for 27% of total DALYs of all cancers in women [24]. The availability of cancer screening is limited to city centres, thus limiting the access to cancer screening. People from rural areas cannot access those facilities and are possibly living with undiagnosed cancer cases, besides, there are large regional variations [25–28]. The Government of India has acknowledged cancer screening as a key strategy for reducing disease burden. The guidelines came into existence in 2016 and recommended to screen for the breast and cervical cancer among women aged 30 years and above [15]. Provision for breast cancer screening have been made at the subcentres and primary health centres (PHC) and the positive cases are referred to district hospital (DH) or community health centre (CHC). For suspicious or malignant lump, provision of biopsy have been made at DH or at CHC, and the cancer cases are referred to medical colleges or tertiary cancer care (TCC). Similarly, in case of cervical screening, women are screened at PHC by visual inspection using acetic acid (VIA). Women with positive VIA are referred to PHC or CHC or DH wherever a lady medical professional is available and if biopsy report indicates cancer, then they are referred to medical colleges or TCC [15]. There are limited empirical population-based studies on the extent of breast and cervical cancers screening in India. In this context, the aim of this study is to examine the socio-economic and regional variations in screening for breast and cervical cancers among Indian women in recommended age (30-49 years) and women in reproductive age (15-49 years). This study is important as it maps the target areas and vulnerable groups that need special focus to increase the currently low screening participation, particularly for breast and cervical cancers among women in the childbearing and economically productive ages.

## Data & methods

### Data

We used unit level data from the most recent round of the nationally representative National Family Health Survey of India 2019-21, i.e., NFHS-5, conducted by the International Institute for Population Sciences, Mumbai under the stewardship of the Ministry of Health and Family Welfare, Government of India. The aim of the survey was to provide reliable data on maternal and child health indicators, nutrition, health service utilization, contraception use and disease screening along with the socio-demographic and economic conditions of households across the country [29]. NFHS-5 used a multistage stratified sampling as part of which the census enumeration blocks (CEBs) in urban areas and villages in rural areas were the primary sampling units (PSUs). Probability Proportional to Size (PPS) sampling was used to select the PSUs. The content and coverage of the survey have widened over time. In NFHS 5, the questions on screening for and diagnosis of cancer were asked to women aged 15–49 years. The survey mainly focused on collecting information on self-reported screening (ever) of three cancers among women: cervical breast, and oral cavity. In NFHS-5, a total of 636,699 households, 724,115 women aged 15–49 and 101,839 men aged 15–54 were interviewed. The sampling design and findings of the survey are publicly available in the report [30]. As the screening for breast and cervical cancer is recommended for women aged 30 years and above, we have used a sample of 357,353 women of 30 to 49 years in the analysis. We have also extended the analyses to 707,119 women in reproductive age and provided these results in supplementary materials (Additional file 1).

### Outcome variables

Self-reported breast cancer and cervical cancer screening were the two main outcome variables. These two variables were recorded in the binary format as “Yes” and “No”. Along with, these we have considered another two outcome variables ever screened for either breast or cervical cancer (yes = 1, no = 0) and ever screened for both breast and cervical cancer (yes = 1, no = 0).

### Independent variables

Based on the previous literatures, a set of 15 independent variables were used [6, 31, 32]. While some of the variables were at the individual level (women specific), others were related to households. The variables relating to women were age, marital status, religion, social group, place of residence, health insurance, use of hormonal contraception, body-mass index (BMI), drinking habits, tobacco consumption, eating habits, regions and education. Household economic condition was measured using the wealth index. The wealth index is a composite variable computed from a set of consumer durables (car, refrigerator, television, mobile etc.), household amenities (drinking water, toilet facility, source of drinking water) and materials used for constructing the house and has been extensively used in literature [30]. The wealth scores were generated using the principal component analysis, separately for rural and urban areas. The households were ranked on the wealth score and the population was divided into five equal categories (poorest, poorer, middle, richer, and richest) where each category contained 20% of the population. The detailed methodology used to derive the wealth index is available on the official website of the Demographic and Health Survey (DHS) [33].

### Statistical analysis

Descriptive statistics, Concentration Index (CI), Concentration Curve (CC), and Logistic regression were used in the analysis. The proportion of breast and cervical cancer screening in India was very low and hence, screening proportions were estimated per 100,000 women. The statistical analysis was done using STATA 17 version.

### Concentration index and concentration curve

Concentration index (CI) and Concentration curve (CC) were used to examine the socio-economic inequality in breast and cervical cancer screening. CC was used to plot the cumulative proportion of the women (ranked by wealth) against the cumulative proportions of the women utilizing breast and cervical cancer screening facilities. If CC and line of equality overlap, then the utilization of breast and cervical cancer screening facilities is evenly distributed across the wealth group. However, if CC lies above the line of equality, it implies a pro-poor concentration of utilization of breast and cervical cancer screening. In contrast, if CC lies below the line of equality, it shows a pro-rich concentration of utilization of breast and cervical cancer screening. On the other hand, CI is defined as twice the area between the CC and the line of equality. The value of CI ranges from − 1 to + 1, with a value of zero suggesting an equal distribution of breast and cervical cancer screening across the wealth group. A negative value signifies a pro-poor distribution of cancer screening, while a positive value signifies a pro-rich distribution [34].

### Logistic regression

A set of four logistic regressions were carried out to determine the significant predictors of breast and cervical cancer screening among Indian women. Outcome variables were ever screened for breast cancer (yes = 1, no = 0), ever screened for cervical cancer (yes = 1, no = 0), ever screened for either breast or cervical cancer (yes = 1, no = 0) and ever screened for both breast and cervical cancer (yes = 1, no = 0). The model specification is given below:

$${ln}\left({Y}_{i}\right)=\alpha +\sum _{i=1}^{n}{\beta }_{i}{X}_{i}$$

Where Y_i_ is the binary outcome variable, mentioned above, β_i_ is the i-th co-efficient, X_i_ is the i-th independent variable and $\alpha$ is the intercept term.

## Results

Table 1 presents the sample characteristics of the study women aged 30–49 years. More than half of the women in the sample were 30 to 39 years of age. The majority of the women were married (91%) and belonged to the Hindu religion (82%). About two-thirds of the respondents resided in rural areas and only 34% of the women had any health insurance. The majority of the women had secondary education (39%) and only 10% had higher secondary and above level of education. A total of 17% of the households had a female household head. Table A1 of additional file shows the full sample of 15 to 49 years of women.

**Table 1:** Sample characteristics of the study women aged 30–49 years, India, 2019–21

Socio economic variables	Percent	Sample size (N)
**Age group**		
30–39	54.2	195,158
40–49	45.8	162,195
**Marital status**		
Married	91.0	323,923
Others	9.0	33,430
**Religion**		
Hindu	82.3	271,320
Muslim	12.1	40,352
Christian	2.6	26,913
Others	3.0	18,768
**Caste**		
SC	21.2	66,434
ST	9.1	66,777
OBC	42.9	136,093
Others	26.9	88,049
**Residence**		
Urban	33.9	92,574
Rural	66.1	264,779
**Health insurance**		
No	65.6	227,413
Yes	34.4	129,940
**Wealth quintile**		
Poorest	17.8	72,074
Poorer	19.1	76,424
Middle	20.5	74,540
Richer	21.2	69,800
Richest	21.3	64,515
**Ever used hormonal contraception**		
No	85.4	299,873
Yes	14.6	57,480
**BMI**		
Thin	10.4	36,856
Normal	55.3	204,331
Overweight or obese	34.2	112,333
**Drink alcohol**		
No	98.9	347,648
Yes	1.1	9705
**Tobacco use**		
No	93.6	323,203
Yes	6.5	34,150
**Eat fried food**		
Never	4.9	17,884
Daily	7.3	32,999
Weekly	34.3	116,602
Occasionally	53.6	189,868
**Eat fruits**		
Never	1.9	5807
Daily	12.1	41,418
Weekly	36.7	130,620
Occasionally	49.4	1,79,508
**Education**		
No education	35.4	130,054
Primary	15.5	55,241
Secondary	38.7	139,755
Higher secondary and above	10.4	32,303
**Sex of the household head**		
Male	83.5	298,456
Female	16.5	58,897
**Media exposure**		
No	26.4	99,969
Yes	73.6	257,384
**Region**		
North	13.9	72,192
Central	22.4	76,015
East	21.9	55,598
Northeast	3.8	52,928
West	14.8	37,811
South	23.2	62,809
**Total**	**100.0**	**357,353**

The socio-economic variations in the proportion of breast and cervical cancer screening per 100,000 women aged 30–49 years are shown in Table 2. The proportion of cancer screening increased with women’s age. For instance, the proportion of screening for breast cancer was 799 among women aged 30–39 compared to 969 among women aged 40–49. The pattern was similar in the case of cervical screening but was of a higher magnitude. The proportion of screening for breast and cervical cancer was significantly higher among married women, being 879 for breast cancer and 1972 for cervical cancer. Women belonging to the Christian religion had a higher proportion of screening for both cervical and breast cancers. The proportion of cancer screening had a strong economic gradient. The screening for breast cancer was 378 among women in the poorest wealth quintile compared to 1331 among women in the richest wealth quintile. The pattern was similar for cervical cancer. The estimated proportion of screening for breast cancer among women with an educational level of higher secondary and above was 1559 and for cervical cancer, it was 2448. On the other hand, women with no education had a lower screening proportion (442 for breast cancer and 1425 for cervical cancer). Regional variation in the proportion of cancer screening did exist. It was observed that the southern and western regions had a significantly higher proportion of screening than the other regions. Table A2 in the additional file shows the socio-economic variations of screening among women aged 15 to 49 years.

**Table 2:** Socio-economic variations in the proportion of breast and cervical cancer screening among women aged 30–49 years (Per 100,000 women) in India, 2019–21

Socio economic factors	Breast cancer	Cervical cancer	Either breast or cervical	Both breast & cervical	Sample Size (N)
**Age group**					
30–39	799	1722	1919	602	195,158
40–49	969	2253	2483	739	162,195
**Marital status**					
Married	879	1972	2187	665	323,923
Others	851	1895	2079	668	33,430
**Religion**					
Hindu	905	2006	2222	690	271,320
Muslim	534	1156	1344	346	40,352
Christian	1337	3752	4079	1011	26,913
Others	1086	2579	2692	972	18,768
**Caste**					
SC	1010	2348	2541	817	66,434
ST	399	942	1053	288	66,777
OBC	1083	2339	2605	817	136,093
Others	604	1411	1586	429	88,049
**Residence**					
Urban	1260	2348	2675	933	92,574
Rural	680	1769	1922	527	264,779
**Health insurance**					
No	893	1876	2062	707	227,413
Yes	846	2135	2397	584	129,940
**Wealth index**					
Poorest	378	990	1095	273	72,074
Poorer	674	1624	1772	526	76,424
Middle	905	2227	2419	713	74,540
Richer	995	2372	2597	771	69,800
Richest	1331	2430	2796	965	64,515
**Ever used hormonal contraception**					
No	954	2155	2379	730	299,873
Yes	426	859	998	286	57,480
**BMI**					
Thin	663	1564	1697	529	36,856
Normal	662	1615	1784	493	204,331
Overweight or obese	1296	2697	3001	992	112,333
**Drink alcohol**					
No	885	1973	2186	671	347,648
Yes	156	1284	1375	65	9705
**Tobacco use**					
No	909	2027	2243	692	323,203
Yes	418	1076	1217	276	34,150
**Eat fried food**					
Never	1145	2535	2758	923	17,884
Daily	496	1356	1555	298	32,999
Weekly	945	2016	2245	715	116,602
Occasionally	861	1964	2166	659	189,868
**Eat fruits**					
Never	468	1065	1221	311	5807
Daily	1193	2362	2725	831	41,418
Weekly	1044	2206	2435	814	130,620
Occasionally	691	1723	1887	526	179,508
**Education**					
No education	442	1425	1542	324	130,054
Primary	883	2095	2246	732	55,241
Secondary	1089	2278	2536	830	139,755
Higher secondary and above	1559	2448	2898	1109	32,303
**Household head's sex**					
Male	859	1954	2165	649	298,456
Female	965	2020	2240	745	58,897
**Media exposure**					
No	389	1175	1273	291	99,969
Yes	1051	2248	2501	799	257,384
**Region**					
North	250	898	1004	144	72,192
Central	402	1273	1374	301	76,015
East	223	559	650	132	55,598
Northeast	362	561	751	172	52,928
West	967	1708	1819	857	37,811
South	2352	4991	5556	1787	62,809
**India**	**877**	**1965**	**2177**	**665**	**357,353**

**Table 3:** State pattern of breast and cervical cancer screening proportion among women aged 30–49 years (Per 100,000 women) in India, 2019–21

State^a^	Breast cancer	Cervical cancer	Either breast or cervical	Both breast & cervical	Sample
**North**					
Delhi	304	711	823	193	5457
Haryana	303	796	883	216	10,831
Himachal Pradesh	433	885	1130	188	6090
Jammu & Kashmir	283	476	543	216	10,787
Punjab	337	2578	2675	240	11,571
Rajasthan	170	415	518	66	19,416
**Central**					
Madhya Pradesh	544	849	872	522	22,546
Uttar Pradesh	379	1590	1718	251	39,893
Chhattisgarh	212	287	398	100	13,576
**East**					
Odisha	213	923	1003	133	14,460
West Bengal	159	199	291	67	10,880
Bihar	341	838	955	224	18,013
Jharkhand	109	470	495	84	12,245
**North-East**					
Arunachal Pradesh	335	848	953	230	10,282
Assam	192	210	303	99	17,545
Manipur	1569	2155	3354	370	4390
Mizoram	2723	7041	8039	1724	4029
**West**					
Gujarat	137	247	297	87	17,389
Maharashtra	1384	2462	2595	1251	17,923
**South**					
Karnataka	362	543	740	165	16,221
Telangana	352	3431	3614	169	14,930
Andhra Pradesh	786	4736	5148	375	6171
Kerala	2429	3530	4629	1330	6631
Tamil Nadu	5781	10,078	10,945	4913	14,655
**India**	**877**	**1965**	**2177**	**665**	**357,353**

^a^Removed other states due to smaller sample size

Table 3 presents the state pattern of breast and cervical cancer screening per 100,000 women aged 30–49 years in India, 2019-21. Breast cancer screening was the highest in Tamil Nadu (5781), followed by Mizoram (2723) and Kerala (2429) and it was the lowest in the states of Jharkhand (109) followed by Gujarat (137) and West Bengal (159). In case of cervical cancer, overall, 1965 women had ever undergone the screening. Cervical cancer screening was also highest in Tamil Nadu (10,078) and it was lowest in West Bengal (199). The state pattern for screening among women aged 15 to 49 is shown in Table A3 of additional file.

**Table 4:** Concentration Index (CI) for breast and cervical cancer screening among women aged 30–49 years by regions of India, 2019–21

**Region**	**Sample size (N)**	**Concentration index**	***P*-value**
**Breast cancer screening**			
North	72,192	0.23	0.001
Central	76,015	0.08	0.105
East	55,598	0.05	0.505
North-East	52,928	0.39	0.000
West	37,811	0.24	0.015
South	62,809	0.07	0.018
**India**	**357,353**	**0.20**	**0.000**
**Cervical cancer screening**			
North	72,192	0.27	0.000
Central	76,015	-0.03	0.303
East	55,598	0.04	0.285
North-East	52,928	0.40	0.000
West	37,811	0.14	0.027
South	62,809	0.02	0.253
**India**	**357,353**	**0.15**	**0.000**
**Either breast or cervical**			
North	72,192	0.27	0.000
Central	76,015	-0.02	0.431
East	55,598	0.05	0.166
North-East	52,928	0.38	0.000
West	37,811	0.14	0.021
South	62,809	0.04	0.034
**India**	**357,353**	**0.16**	**0.000**
**Both breast & cervical**			
North	72,192	0.23	0.002
Central	76,015	0.08	0.190
East	55,598	0.00	0.953
North-East	52,928	0.44	0.000
West	37,811	0.25	0.020
South	62,809	0.03	0.371
**India**	**357,353**	**0.20**	**0.029**

Table 4 presents the concentration index (CI) for breast and cervical cancer screening by the regions of India, 2019-21. The overall CI value was 0.2 for breast cancer screening and 0.15 for cervical cancer screening, suggesting a pro-rich utilization of breast and cervical cancer screening in India. The CI value for each region indicates that the utilization of breast cancer screening was pro-rich and was significantly highest in the north-eastern region than the other regions and was the lowest in the southern region. The pattern was similar for cervical cancer screening. Similar trend has been observed in case of the women aged 15 to 49 years (additional table A4).

**Table 5:** Odds ratio (OR) and 95% confidence interval (CI) for uptaking breast and cervical cancer screening among women aged 30–49 years in India, 2019–21

Socio-economic and risk factors	Breast		Cervical		Either breast or cervical		Both breast & cervical	
	**OR**	**95% CI**	**OR**	**95% CI**	**OR**	**95% CI**	**OR**	**95% CI**
**Age group**								
30–39®	1.00		1.00		1.00		1.00	
40–49	1.35^c^	[1.24, 1.47]	1.36^c^	[1.29, 1.44]	1.35^c^	[1.28, 1.43]	1.38^c^	[1.25, 1.53]
**Education**								
No education ®	1.00		1.00		1.00		1.00	
Primary	1.53^c^	[1.31, 1.77]	1.21^c^	[1.11, 1.32]	1.19^c^	[1.1, 1.3]	1.73^c^	[1.45, 2.05]
Secondary	2.04^c^	[1.8, 2.32]	1.3^c^	[1.21, 1.4]	1.34^c^	[1.25, 1.44]	2.15^c^	[1.85, 2.49]
Higher secondary and above	2.68^c^	[2.26, 3.18]	1.36^c^	[1.22, 1.52]	1.46^c^	[1.31, 1.62]	2.71^c^	[2.21, 3.31]
**Marital status**								
Others ®	1.00		1.00		1.00		1.00	
Married	1.18^b^	[1.02, 1.38]	1.21^c^	[1.09, 1.35]	1.22^c^	[1.1, 1.35]	1.14	[0.95, 1.38]
**Health insurance**								
No ®	1.00		1.00		1.00		1.00	
Yes	0.75^c^	[0.69, 0.83]	0.88^c^	[0.83, 0.93]	0.89^c^	[0.84, 0.94]	0.69^c^	[0.62, 0.77]
**Ever used hormonal contraception**								
No ®	1.00		1.00		1.00		1.00	
Yes	0.95	[0.82, 1.1]	0.83^c^	[0.76, 0.91]	0.86^c^	[0.79, 0.94]	0.88	[0.74, 1.05]
**BMI**								
Thin ®	1.00		1.00		1.00		1.00	
Normal	0.99	[0.83, 1.17]	1.02	[0.92, 1.14]	1.02	[0.92, 1.13]	0.98	[0.81, 1.19]
Overweight or obese	1.38^c^	[1.16, 1.65]	1.3^c^	[1.17, 1.45]	1.31^c^	[1.18, 1.45]	1.42^c^	[1.16, 1.73]
**Drink alcohol**								
No ®	1.00		1.00		1.00		1.00	
Yes	0.48^c^	[0.32, 0.72]	0.79^b^	[0.65, 0.97]	0.76^c^	[0.63, 0.92]	0.47^c^	[0.29, 0.77]
**Tobacco use**								
No ®	1.00		1.00		1.00		1.00	
Yes	1.59^c^	[1.36, 1.87]	1.33^c^	[1.2, 1.47]	1.35^c^	[1.22, 1.49]	1.61^c^	[1.34, 1.95]
**Eat fried food**								
Never ®	1.00		1.00		1.00		1.00	
Daily	0.82	[0.65, 1.02]	1.08	[0.94, 1.25]	1.06	[0.93, 1.22]	0.78	[0.6, 1.01]
Weekly	0.84	[0.71, 1.01]	0.87^b^	[0.77, 0.98]	0.88^b^	[0.79, 0.99]	0.79^b^	[0.65, 0.97]
Occasionally	0.78^c^	[0.66, 0.92]	0.83^c^	[0.74, 0.93]	0.83^c^	[0.75, 0.93]	0.75^c^	[0.62, 0.9]
**Eat fruits**								
Never ®	1.00		1.00		1.00		1.00	
Daily	1.01	[0.68, 1.51]	1.11	[0.86, 1.44]	1.08	[0.84, 1.37]	1.12	[0.69, 1.82]
Weekly	1.06	[0.72, 1.57]	1.12	[0.87, 1.44]	1.07	[0.84, 1.36]	1.24	[0.77, 1.99]
Occasionally	1.09	[0.74, 1.61]	1.23	[0.96, 1.58]	1.17	[0.93, 1.49]	1.25	[0.78, 2]
**Household head's sex**								
Male ®	1.00		1.00		1.00		1.00	
Female	1.02	[0.91, 1.16]	0.98	[0.9, 1.06]	0.98	[0.91, 1.06]	1.03	[0.89, 1.18]
**Religion**								
Hindu ®	1.00		1.00		1.00		1.00	
Muslim	0.77^c^	[0.66, 0.91]	0.7^c^	[0.63, 0.77]	0.72^c^	[0.65, 0.79]	0.72^c^	[0.59, 0.87]
Christian	1.41^c^	[1.2, 1.65]	1.67^c^	[1.5, 1.85]	1.6^c^	[1.44, 1.76]	1.58^c^	[1.31, 1.9]
Others	1.29^b^	[1.01, 1.64]	1.91^c^	[1.68, 2.18]	1.84^c^	[1.63, 2.09]	1.27	[0.93, 1.72]
**Caste**								
SC ®	1.00		1.00		1.00		1.00	
ST	0.7^c^	[0.59, 0.83]	0.66^c^	[0.59, 0.74]	0.65^c^	[0.59, 0.73]	0.74^c^	[0.61, 0.9]
OBC	0.84^c^	[0.75, 0.94]	0.84^c^	[0.78, 0.9]	0.85^c^	[0.8, 0.91]	0.8^c^	[0.7, 0.9]
Others	0.68^c^	[0.59, 0.78]	0.75^c^	[0.69, 0.82]	0.76^c^	[0.7, 0.83]	0.6^c^	[0.51, 0.71]
**Residence**								
Rural ®	1.00		1.00		1.00		1.00	
Urban	1.34^c^	[1.21, 1.48]	1.14^c^	[1.06, 1.21]	1.16^c^	[1.09, 1.23]	1.32^c^	[1.18, 1.49]
**Wealth index**								
Poorest ®	1.00		1.00		1.00		1.00	
Poorer	0.96	[0.8, 1.14]	1.08	[0.97, 1.2]	1.08	[0.97, 1.19]	0.94	[0.77, 1.16]
Middle	0.96	[0.8, 1.15]	1.18^c^	[1.06, 1.32]	1.17^c^	[1.05, 1.3]	0.94	[0.77, 1.16]
Richer	0.8^b^	[0.66, 0.97]	1.12^a^	[1.03, 1.26]	1.11^a^	[0.99, 1.24]	0.75^b^	[0.6, 0.94]
Richest	0.9	[0.73, 1.11]	1.23^c^	[1.08, 1.41]	1.23^c^	[1.08, 1.39]	0.83	[0.65, 1.06]
**Media exposure**								
No ®	1.00		1.00		1.00		1.00	
Yes	1.07	[0.94, 1.22]	1.01	[0.93, 1.09]	1.02	[0.94, 1.1]	1.04	[0.89, 1.21]
**Region**								
East ®	1.00		1.00		1.00		1.00	
North	1.13	[0.88, 1.45]	1.22^c^	[1.06, 1.41]	1.21^c^	[1.06, 1.39]	1.11	[0.81, 1.52]
Central	1.76^c^	[1.41, 2.21]	1.91^c^	[1.68, 2.18]	1.82^c^	[1.61, 2.06]	2.14^c^	[1.62, 2.81]
Northeast	1.68^c^	[1.3, 2.16]	1.24^c^	[1.06, 1.46]	1.38^c^	[1.19, 1.6]	1.27	[0.92, 1.75]
West	2.39^c^	[1.88, 3.04]	1.66^c^	[1.42, 1.93]	1.62^c^	[1.4, 1.87]	3^c^	[2.25, 4]
South	8.47^c^	[6.88, 10.42]	6.82^c^	[6.02, 7.71]	6.66^c^	[5.93, 7.49]	10.29^c^	[7.97, 13.29]

Level of significance:

^c^ < 0.001

^b^ < 0.01

^a^ < 0.05

Table 5 presents the results of logistic regression on determinants of up taking breast and cervical cancer screening among women aged 30 to 49 years in India. The odds of up taking breast and cervical cancer screening had strong age and education gradient. For instance, women with 40 to 49 years of age had significantly higher odds of up taking breast (OR: 1.35; 95% CI: 1.24–1.47) as well as cervical (OR:1.36, 95% CI:1.29–1.44) cancer screening. Similarly, the likelihood of up taking breast and cervical cancer screening was higher among women with higher secondary and above education level than the uneducated women (for breast OR: 2.68; 95% CI: 2.26–3.18 and for cervical OR: 1.36; 95% CI: 1.22–1.52). The odds of breast and cervical cancer screening was also higher among urban women and among women from west and south region.

## Discussion

Despite the growing burden of cancer in India, there are very few nationally representative studies that examine the socio-economic variations in cancer screening among women aged 30–49 years. This age group has higher concentration of women in recommended ages (30 years and above) by Government of India. They are also the major economically productive age group in the population. Given the early onset of NCDs in India and guidelines that provision of cancer screening at public health centers, understanding the status of breast and cervical cancer screening would help evidence based planning. The present study aims to measure the proportion of breast and cervical cancer screening and analyse the socio-economic and regional inequality in its uptake in India among women in the reproductive age using the most recent round of nationally representative survey. The following are the salient findings of this study. First, the overall proportion of breast and cervical screening among women in the 30–49 years of age in India was 877 and 1965 per 100,000 women respectively, lower than in many developing countries. However, it was higher than all women aged 15–49 (additional file table A2). Our results suggest that screening has a strong economic, social and age gradient. Women who belonged to female headed households, belonged to Christian religion, used tobacco products, were overweight, were married and resided in urban areas had a higher uptake of screening for breast or cervical cancer. The pattern was similar for both cancers; however, the screening was lower for breast cancer than cervical cancer. Second, the state and regional variations in cancer screening are high in India. The overall proportion of screening for breast and cervical cancer is higher in southern (Andhra Pradesh, Tamil Nadu, Kerala, Telangana), western (Maharashtra), and some north-eastern states (Mizoram and Manipur) than in the rest of the states of the country. Third, the socio-economic inequality in breast and cervical cancer screening among women aged 30–49 and all women in the reproductive age was pro-rich. At the national level, the concentration index for women aged 30–49 was 0.2 for breast cancer and 0.15 for cervical cancer screening. The socio-economic inequality in cancer screening was lower in the southern region compared to the other regions. Fourth, the result of the multivariate analysis confirmed that women from the southern region had higher log count of screening test for either of the two cancers compared to the women from the remaining regions. The results also confirmed that the chances of undergoing breast and cervical cancer screening were higher in the urban areas, those with higher level of education, those who were married and those who were older.

We have some plausible explanations for the above results. Despite continuous governmental efforts from introducing cancer screening and awareness programs starting with the launch of the National Cancer Control Programme in 1975 to launching the National Programme for Prevention and Control of Cancer, Diabetes, Cardiovascular Diseases and Stroke (NPCDCS) by the Ministry of Health and Family Welfare (MoHFW) in 2010, the screening for breast and cervical cancer among women has continued to remain low. At the same time, mortality due to breast and cervical cancers remains the highest in the country [26]. The NPCDCS aims to prevent and control chronic NCDs, including cancer, through opportunistic screening and/or using the camp approach at different levels of health facilities among the population aged 30 years and above [35]. In 2012, the Government of India formed the National Cancer Grid of India (NCG) with the aim of setting uniform standards of patient care in India through evidence-based cancer prevention, screening and management guidelines [36]. The Indian government published the country’s first cancer screening operational framework in 2016, which aims to provide mandatory cancer screening for cervical, breast and oral cancers for the population over 30 years of age in 100 districts using a cost-effective methodology [15]. However, these guidelines have not been executed effectively in most of the states. Previous literature suggests that breast and cervical cancer examination is higher among women aged 25 to 39 years within the overall reproductive age-group [6]. However, our study showed that screening uptake was significantly higher among women in the 30 to 39 years and 40 to 49 years age groups.

Breast cancer is easier to diagnose than the other women’s cancers yet, the screening for it is one of the lowest even though the disease is prevalent across the country [37]. One possible reason for the lower screening of breast cancer compared to cervical cancer may be the lack of opportunistic screening [38]. When women avail reproductive healthcare facilities or go for any gynaecological issues, the concerned physicians often refer them for cervical cancer screening. By contrast, no such opportunistic screening programmes are available for breast cancer in India [39, 40]. At present, women mostly go for screening when the symptoms have already developed. The average cost of breast or cervical screening varies by type of health centre and across states. For instance, in a leading public hospital in Mumbai, the average cost of cancer screening was INR 5000 (USD 63). In rural areas, where over two-thirds of the population resides, the accessibility to cancer screening is limited.

The lower proportion of breast and cervical cancer screening in the 15 to 49 years age group in India can be explained from two major perspectives: first, the lack of necessary health infrastructure in the three-tier system and screening programmes, and second, the socio-cultural beliefs and economic factors. Despite the higher share of breast and cervical cancers among all cancers in the country, a robust national level screening programme is missing. Mammography and ultrasound scan (USS) are two sensitive breast cancer screening procedures in India. Although mammogram has a sensitivity of 62–68% and is ineffective in women with dense breast tissues and women below 35 years of age, the scarcity of mammograms in rural India leads to delay in diagnosis as well as treatment [41]. This is one of the reasons that almost 70% of all breast cancer cases present in the advanced stages when the treatment options are very limited [42]. On the other hand, even though USS is more sensitive and effective in women aged below 35 years, it cannot be used as a community-based screening tool due to the Pre-Conception and Pre-Natal Diagnostic Techniques (PCPNDT) Act, 1994 that aims to prevent female feticide [42]. Apart from that, USS warrants the test to be conducted by medical professionals, of whom there is a scarcity in the remote settings [41]. For almost the same set of reasons, cervical cancer screening is also low among Indian women. Apart from visual inspection with acetic acid (VIA), the other two screening modalities for cervical cancer, that is, cytology (Pap smear) and Human Papillomavirus Test (HPV test) require trained medical attendees along with a sophisticated laboratory infrastructure which are only available in metro-city centric health facilities [28].

Apart from the lack of health infrastructure and national screening programmes, the socio-economic and cultural factors relating to breast and cervical cancer screening also play a prominent role. Most of the time in the early stage of breast cancer, patients feel a painless lump in the breast. However, women from the lower socio-economic sections, having lower incomes and those with low education are unaware of this symptom of breast cancer [41]. Studies have also identified stigma of rejection by the community or a partner, fear of loss of breasts, taboo of not discussing breast cancer openly, embarrassment revealing body parts, especially to male healthcare providers, fatalistic attitude, and lack of family support as the major barriers to the uptake of screening for breast as well as cervical cancer [43, 44].

Education is a significant factor in the uptake of any cancer screening among women in the reproductive age-group. Our study demonstrates that women with higher levels of education have a higher uptake of screening. This finding is similar to the findings of other studies on screening in the developing countries [45, 46]. It is also observed that female headed households have a strong influence on breast and cervical cancer screening. A study suggests that female headed households are more likely to recognize reproductive health issues of women that are unique to women [6]. Recognizing the problems and getting the right treatment is a major driving force to increase cancer screening. Another reason may be the fact that female headed households generally have a better opportunity for healthcare decision making [41].

There are some limitations of our study. First, our analysis was restricted to women aged 15–49 years with emphasis on 30–49 because the NFHS provides data for this age group only. Consequently, we could not analyze cancer screening among women aged 50 years and above. Second, the NFHS provides data on self-reported ever screening which may be subject to self-reporting biases and reporting errors. Moreover, the most recent screening activity could not be segregated and questions on time of cancer screening were not canvassed. Third, it was not possible to differentiate between women who had undergone screening for preventive purposes and those who had undergone it after developing the disease due to the non-availability of data.

## Conclusion

Breast and cervical cancers are a growing public health concern among women in India. Apart from socio-economic factors, other factors like lack of screening infrastructure, lack of awareness, associated stigma, and taboos are important correlates of the lower uptake of cancer screening. Despite the operational guideline and provisioning screening at public health centres, the screening uptake is low in the country. A high-quality national screening programme for women’s cancer comprising women health care professionals, with high coverage and participation and an effective referral system is very much required to change the current scenario. Providing knowledge on self-breast examination (SBE) and self-awareness can be a key strategy along with infrastructural improvements. Trained community health workers may help to overcome the stigma and taboos associated with breast and cervical cancers.

**Fig. 1:**
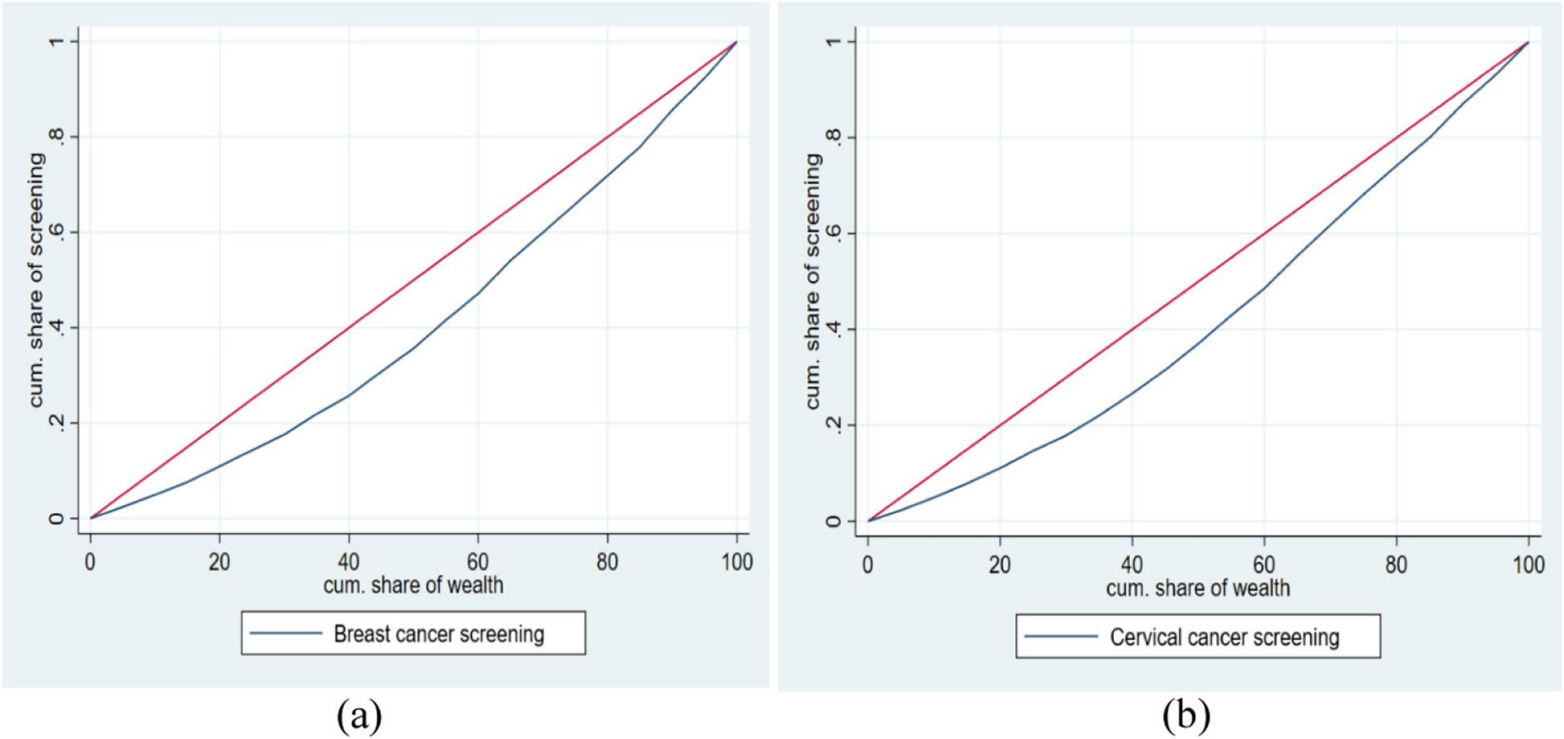
Concentration curve for breast and cervical cancer screening among women aged 30–49 years in India, 2019–2021. Figures 1 (**a**) and (**b**) present the concentration curves (CC) for breast and cervical cancer screening, among women in the 30 to 49 years age group. The CC for women who had undergone breast cancer screening was below the line of equality, suggesting a pro-rich concentration of breast cancer screening. The pattern of CC was similar for cervical cancer screening indicating a pro-rich concentration of cervical cancer screening

## Supplementary Information

**Additional file 1:** **Table A1.** Sample characteristics of the study women aged 15-49 years, India, 2019-21. **Table A2.** Socio-economic differential in the proportion of breast and cervical cancer screening among women aged 15-49 years (Per 100,000 women) in India, 2019-21. **Table A3.** State pattern of breast and cervical cancer screening proportion among women aged 15-49 years (Per 100,000 women) in India, 2019-21. **Table A4.** Concentration Index (CI) for breast and cervical cancer screening among women aged 15-49 years by regions of India, 2019-21. **Figure A1.** Concentration curve for breast and cervical cancer screening among women aged 15-49 years in India, 2019-2021.

## Data Availability

The data is publicly available from https://dhsprogram.com/data/dataset/India_Standard-DHS_2020.cfm?flag=0 .

## Abbreviations

DALY: Disability Adjusted Life Years
YLL: Years of Life Lost
NCD: Non-Communicable Disease
LMIC: Low and Middle Income Country
NFHS: National Family Health Survey
MoHFW: Ministry of Health and Family Survey
DHS: Demographic and Health Surveys
BMI: Body-Mass Index
CI: Concentration Index
CC: Concentration Curve
OR: Odds Ratio
