# Supplementary material for: Socio-economic and regional variation in breast and cervical cancer screening among Indian women of reproductive age: a study from National Family Health Survey, 2019-21
Source: BMC Cancer. 2022 Dec 7;22:1279. doi: 10.1186/s12885-022-10387-9 (PMC9727878; doi:10.1186/s12885-022-10387-9)
Supplement: Supplementary file 1 — Additional file 1: Table A1. Sample characteristics of the study women aged 15-49 years, India, 2019-21. Table A2. Socio-economic differential in the proportion of breast and cervical cancer screening among women aged 15-49 years (Per 100,000 women) in India, 2019-21. Table A3. State pattern of breast and cervical cancer screening proportion among women aged 15-49 years (Per 100,000 women) in India, 2019-21. Table A4. Concentration Index (CI) for breast and cervical cancer screening among women aged 15-49 years by regions of India, 2019-21. Figure A1. Concentration curve for breast and cervical cancer screening among women aged 15-49 years in India, 2019-2021. [file 12885_2022_10387_MOESM1_ESM.docx]

**Table A1**: **Sample characteristics of the study women aged 15-49 years, India, 2019-21.**

| **Socio economic variables** | **Percent** | **Sample Size (N)** |
| --- | --- | --- |
| **Age group** |  |  |
| 15-29 | 49.5 | 349,766 |
| 30-39 | 27.6 | 195,158 |
| 40-49 | 22.9 | 162,195 |
| **Marital status** |  |  |
| Married | 71.1 | 502,467 |
| Others | 28.9 | 204,652 |
| **Religion** |  |  |
| Hindu | 75.5 | 533,521 |
| Muslim | 12.4 | 87,825 |
| Christian | 7.3 | 51,564 |
| Others | 4.8 | 34,209 |
| **Caste** |  |  |
| SC | 19.3 | 136,635 |
| ST | 18.9 | 133,273 |
| OBC | 38.2 | 270,037 |
| Others | 23.6 | 167,174 |
| **Residence** |  |  |
| Urban | 24.5 | 173,171 |
| Rural | 75.5 | 533,948 |
| **Health Insurance** |  |  |
| No | 67.8 | 479,569 |
| Yes | 32.2 | 227,550 |
| **Wealth Quintile** |  |  |
| Poorest | 20.8 | 146,997 |
| Poorer | 22.3 | 157,411 |
| Middle | 21.0 | 148,486 |
| Richer | 19.3 | 136,317 |
| Richest | 16.7 | 117,908 |
| **Ever used hormonal contraception** | | |
| No | 87.7 | 620,049 |
| Yes | 12.3 | 87,070 |
| **BMI** |  |  |
| Thin | 17.9 | 124,931 |
| Normal | 60.1 | 420,298 |
| Overweight or obese | 22.0 | 153,914 |
| **Drink Alcohol** |  |  |
| No | 98.1 | 693,746 |
| Yes | 1.9 | 13,373 |
| **Tobacco use** |  |  |
| No | 93.4 | 660,644 |
| Yes | 6.6 | 46,475 |
| **Eat fried food** |  |  |
| Never | 4.6 | 32,189 |
| Daily | 9.4 | 66,679 |
| Weekly | 33.8 | 238,993 |
| Occasionally | 52.2 | 369,258 |
| **Eat fruits** |  |  |
| Never | 1.5 | 10,269 |
| Daily | 11.9 | 84,249 |
| Weekly | 37.1 | 261,955 |
| Occasionally | 49.6 | 350,646 |
| **Education** |  |  |
| No education | 23.2 | 163,951 |
| Primary | 11.8 | 83,470 |
| Secondary | 51.1 | 361,385 |
| Higher secondary and above | 13.9 | 98,313 |
| **Sex of the household head** | |  |
| Male | 83.9 | 593,430 |
| Female | 16.1 | 113,689 |
| **Media Exposure** |  |  |
| No | 26.1 | 184,321 |
| Yes | 73.9 | 522,798 |
| **Region** |  |  |
| North | 20.3 | 143,303 |
| Central | 23.3 | 164,949 |
| East | 16.4 | 116,264 |
| Northeast | 14.5 | 102,427 |
| West | 9.9 | 70,321 |
| South | 15.5 | 109,855 |
| **India** | **100.0** | **707,119** |

**Table A2:** **Socio-economic differential in the proportion of breast and cervical cancer screening among women aged 15-49 years (Per 100,000 women) in India, 2019-21.**

| **Socio economic factors** | **Breast cancer screening** | **Cervical cancer screening** | **Either breast or cervical screening** | **Both breast & cervical screening** | **Sample size**  **(N)** |
| --- | --- | --- | --- | --- | --- |
| **Age group** | |  |  |  |  |
| 15-29 | 317 | 708 | 782 | 243 | 349,766 |
| 30-39 | 799 | 1722 | 1919 | 602 | 195,158 |
| 40-49 | 969 | 2253 | 2483 | 739 | 162,195 |
| **Marital status** | |  |  |  |  |
| Married | 729 | 1639 | 1810 | 557 | 502,467 |
| Others | 264 | 573 | 644 | 193 | 204,652 |
| **Religion** |  |  |  |  |  |
| Hindu | 621 | 1379 | 1527 | 473 | 533,521 |
| Muslim | 356 | 785 | 891 | 250 | 87,825 |
| Christian | 978 | 2580 | 2868 | 690 | 51,564 |
| Others | 846 | 1974 | 2063 | 757 | 34,209 |
| **Caste** |  |  |  |  |  |
| SC | 664 | 1552 | 1677 | 539 | 136,635 |
| ST | 311 | 675 | 750 | 236 | 133,273 |
| OBC | 725 | 1590 | 1772 | 543 | 270,037 |
| Others | 442 | 999 | 1120 | 321 | 167,174 |
| **Residence** | |  |  |  |  |
| Urban | 873 | 1637 | 1859 | 652 | 173,171 |
| Rural | 472 | 1206 | 1314 | 364 | 533,948 |
| **Health Insurance** | |  |  |  |  |
| No | 597 | 1268 | 1396 | 469 | 479,569 |
| Yes | 607 | 1521 | 1701 | 427 | 227,550 |
| **Wealth Index** | |  |  |  |  |
| Poorest | 274 | 690 | 758 | 205 | 146,997 |
| Poorer | 455 | 1078 | 1176 | 357 | 157,411 |
| Middle | 638 | 1515 | 1665 | 488 | 148,486 |
| Richer | 679 | 1652 | 1803 | 528 | 136,317 |
| Richest | 935 | 1731 | 1980 | 686 | 117,908 |
| **Ever used hormonal contraception** | | | |  |  |
| No | 626 | 1419 | 1564 | 481 | 620,049 |
| Yes | 398 | 760 | 896 | 261 | 87,070 |
| **BMI** |  |  |  |  |  |
| Thin | 342 | 815 | 890 | 267 | 124,931 |
| Normal | 476 | 1122 | 1245 | 354 | 420,298 |
| Overweight or obese | 1102 | 2324 | 2572 | 854 | 153,914 |
| **Drink Alcohol** | |  |  |  |  |
| No | 604 | 1347 | 1491 | 459 | 693,746 |
| Yes | 119 | 973 | 1044 | 48 | 13,373 |
| **Tobacco use** | |  |  |  |  |
| No | 610 | 1362 | 1506 | 466 | 660,644 |
| Yes | 359 | 927 | 1078 | 233 | 46,475 |
| **Eat fried food** | |  |  |  |  |
| Never | 781 | 1762 | 1902 | 640 | 32,189 |
| Daily | 331 | 905 | 1024 | 213 | 66,679 |
| Weekly | 620 | 1368 | 1519 | 470 | 238,993 |
| Occasionally | 610 | 1355 | 1498 | 466 | 369,258 |
| **Eat fruits** | |  |  |  |  |
| Never | 326 | 778 | 891 | 212 | 10,269 |
| Daily | 774 | 1716 | 1930 | 560 | 84,249 |
| Weekly | 726 | 1510 | 1675 | 561 | 261,955 |
| Occasionally | 470 | 1143 | 1255 | 359 | 350,646 |
| **Education** | |  |  |  |  |
| No education | 394 | 1239 | 1346 | 288 | 163,951 |
| Primary | 671 | 1633 | 1751 | 554 | 83,470 |
| Secondary | 602 | 1291 | 1432 | 461 | 361,385 |
| Higher secondary and above | 843 | 1447 | 1676 | 613 | 98,313 |
| **Household head's sex** | | |  |  |  |
| Male | 593 | 1336 | 1478 | 451 | 593,430 |
| Female | 638 | 1382 | 1537 | 483 | 113,689 |
| **Media Exposure** | |  |  |  |  |
| No | 317 | 862 | 937 | 243 | 184,321 |
| Yes | 694 | 1505 | 1672 | 527 | 522,798 |
| **Region** |  |  |  |  |  |
| North | 204 | 666 | 736 | 134 | 143,303 |
| Central | 329 | 909 | 995 | 244 | 164,949 |
| East | 168 | 389 | 454 | 104 | 116,264 |
| Northeast | 264 | 413 | 532 | 145 | 102,427 |
| West | 789 | 1343 | 1431 | 701 | 70,321 |
| South | 1620 | 3583 | 3981 | 1222 | 109,855 |
| **India** | **600** | **1344** | **1488** | **456** | **707,119** |

**Table A3: State pattern of breast and cervical cancer screening proportion among women aged 15-49 years (Per 100,000 women) in India, 2019-21.**

| **State** | **Breast cancer screening** | **Cervical cancer screening** | **Either breast or cervical screening** | **Both breast & cervical screening** | **Sample size** |
| --- | --- | --- | --- | --- | --- |
| **North** |  |  |  |  |  |
| Delhi | 212 | 468 | 549 | 130 | 10,538 |
| Haryana | 241 | 553 | 622 | 172 | 21,204 |
| Himachal Pradesh | 360 | 706 | 865 | 202 | 10,258 |
| Jammu & Kashmir | 224 | 368 | 432 | 160 | 22,528 |
| Punjab | 316 | 2180 | 2240 | 256 | 20,708 |
| Rajasthan | 143 | 317 | 379 | 81 | 42,263 |
| **Central** |  |  |  |  |  |
| Madhya Pradesh | 531 | 764 | 799 | 496 | 46,590 |
| Uttar Pradesh | 281 | 1039 | 1144 | 175 | 90,496 |
| Chhattisgarh | 186 | 365 | 431 | 119 | 27,863 |
| **East** |  |  |  |  |  |
| Odisha | 137 | 662 | 720 | 80 | 27,649 |
| West Bengal | 105 | 138 | 198 | 46 | 21,089 |
| Bihar | 247 | 532 | 614 | 165 | 41,531 |
| Jharkhand | 153 | 380 | 412 | 120 | 25,995 |
| **North-East** |  |  |  |  |  |
| Arunachal Pradesh | 309 | 722 | 824 | 207 | 19,606 |
| Assam | 166 | 221 | 276 | 111 | 34,565 |
| Manipur | 1020 | 1394 | 2180 | 233 | 7994 |
| Mizoram | 1711 | 4150 | 4833 | 1027 | 7200 |
| **West** |  |  |  |  |  |
| Gujarat | 126 | 232 | 279 | 79 | 32,873 |
| Maharashtra | 1130 | 1926 | 2029 | 1027 | 32,797 |
| **South** |  |  |  |  |  |
| Karnataka | 249 | 499 | 637 | 111 | 29,628 |
| Telangana | 314 | 2310 | 2484 | 141 | 26,443 |
| Andhra Pradesh | 597 | 3418 | 3692 | 323 | 10,732 |
| Kerala | 1638 | 2447 | 3217 | 868 | 10,730 |
| Tamil Nadu | 4033 | 7388 | 8021 | 3400 | 25,107 |
| **India** | **600** | **1344** | **1488** | **456** | **707,119** |

**Table A4: Concentration Index (CI) for breast and cervical cancer screening among women aged 15-49 years by regions of India, 2019-21.**

|  | **Breast cancer screening** | | |
| --- | --- | --- | --- |
| **Region** | **Sample Size (N)** | **Concentration Index** | **P-value** |
| North | 143,303 | 0.194 | 0.000 |
| Central | 164,949 | 0.042 | 0.295 |
| East | 116,264 | 0.022 | 0.680 |
| North-East | 102,427 | 0.299 | 0.000 |
| West | 70,321 | 0.201 | 0.011 |
| South | 109,855 | 0.074 | 0.007 |
| **India** | **707,119** | **0.206** | **0.000** |
|  | **Cervical cancer screening** | | |
| **Region** | **Sample Size(N)** | **Concentration Index** | **P-value** |
| North | 143,303 | 0.240 | 0.000 |
| Central | 164,949 | -0.034 | 0.209 |
| East | 116,264 | 0.034 | 0.262 |
| North-East | 102,427 | 0.293 | 0.000 |
| West | 70,321 | 0.134 | 0.015 |
| South | 109,855 | 0.026 | 0.179 |
| **India** | **707,119** | **0.160** | **0.000** |
|  | **Either breast or cervical** | | |
| **Region** | **Sample Size(N)** | **Concentration Index** | **P-value** |
| North | 143,303 | 0.240 | 0.000 |
| Central | 164,949 | -0.024 | 0.343 |
| East | 116,264 | 0.048 | 0.105 |
| North-East | 102,427 | 0.312 | 0.000 |
| West | 70,321 | 0.131 | 0.013 |
| South | 109,855 | 0.042 | 0.020 |
| **India** | **707,119** | **0.166** | **0.000** |
|  | **Both breast & cervical** | | |
| **Region** | **Sample Size(N)** | **Concentration Index** | **P-value** |
| North | 143,303 | 0.172 | 0.005 |
| Central | 164,949 | 0.029 | 0.544 |
| East | 116,264 | -0.047 | 0.472 |
| North-East | 102,427 | 0.237 | 0.006 |
| West | 70,321 | 0.215 | 0.014 |
| South | 109,855 | 0.036 | 0.278 |
| **India** | **707,119** | **0.199** | **0.000** |


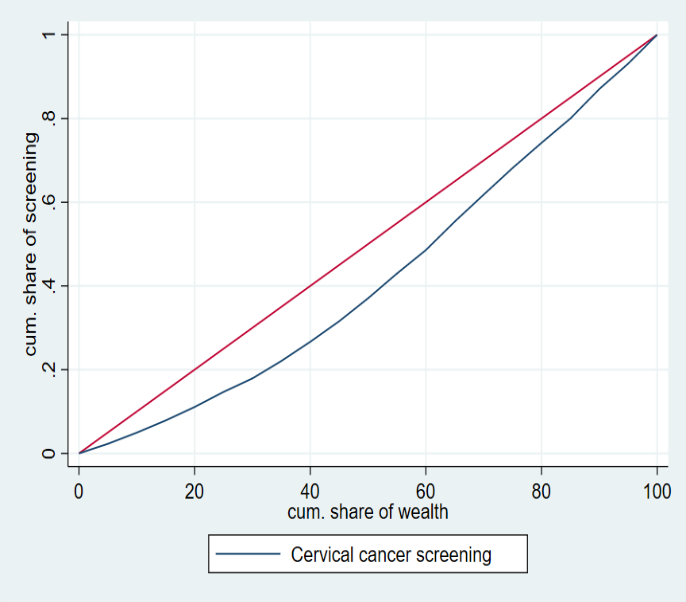

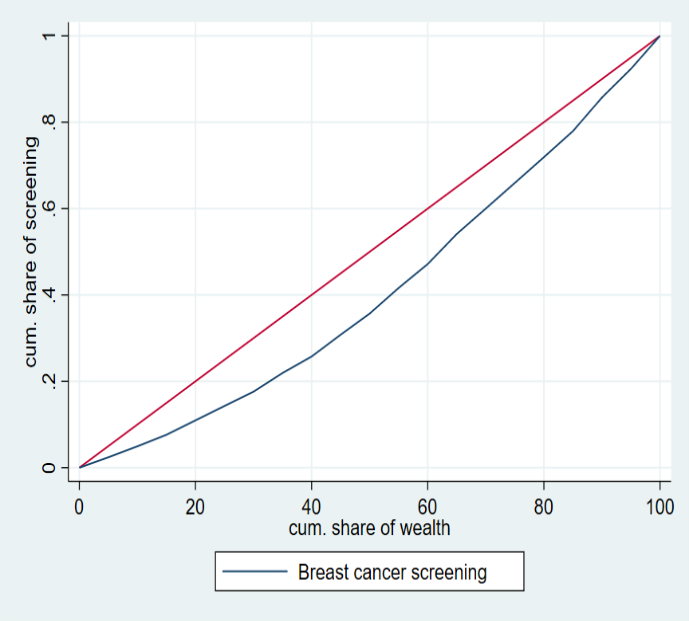
**Figure A1 Concentration curve for breast and cervical cancer screening among women aged 15-49 years in India, 2019-2021**
